# Electromagnetic Field Analysis and Modeling of a Relative Position Detection Sensor for High Speed Maglev Trains

**DOI:** 10.3390/s120506447

**Published:** 2012-05-15

**Authors:** Song Xue, Ning He, Zhiqiang Long

**Affiliations:** College of Mechatronics Engineering and Automation, National University of Defense Technology, Changsha 410073, China; E-Mails: songself@126.com (S.X.); zhqlong@263.net (Z.L.)

**Keywords:** relative position detection sensor, electromagnetic modeling, multipole theory, new equivalent source method, second-order vector potential, difference coil structure

## Abstract

The long stator track for high speed maglev trains has a tooth-slot structure. The sensor obtains precise relative position information for the traction system by detecting the long stator tooth-slot structure based on nondestructive detection technology. The magnetic field modeling of the sensor is a typical three-dimensional (3-D) electromagnetic problem with complex boundary conditions, and is studied semi-analytically in this paper. A second-order vector potential (SOVP) is introduced to simplify the vector field problem to a scalar field one, the solution of which can be expressed in terms of series expansions according to Multipole Theory (MT) and the New Equivalent Source (NES) method. The coefficients of the expansions are determined by the least squares method based on the boundary conditions. Then, the solution is compared to the simulation result through Finite Element Analysis (FEA). The comparison results show that the semi-analytical solution agrees approximately with the numerical solution. Finally, based on electromagnetic modeling, a difference coil structure is designed to improve the sensitivity and accuracy of the sensor.

## 1. Introduction

**Figure 1.**
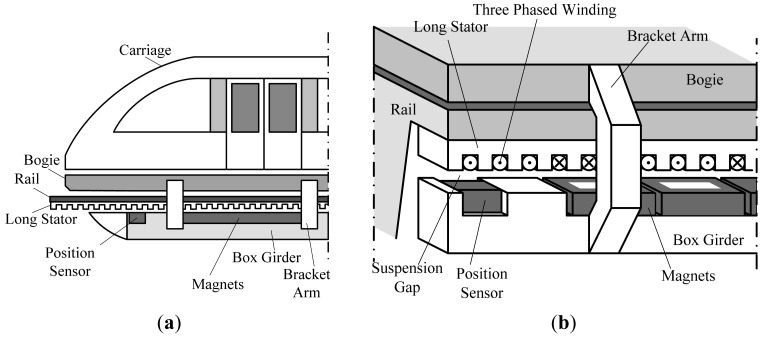
(**a**) Diagram of a high speed maglev train; (**b**) Diagram of the substructure of a high speed maglev train.

High speed maglev trains are driven by a linear synchronous motor [1,2]. The rotor is the suspension electromagnet, and the long stator inlaid with 3-phase windings has a tooth-slot structure [1,3], shown in Figure 1.

**Figure 2.**
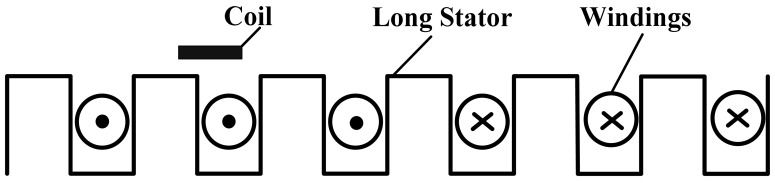
Diagram of the sensor coil and the long stator.

The operating principle of the relative position detection sensor is based on nondestructive examination technology [4–7]. There are detecting coils arranged on one side of the sensor facing the tooth-slot structure, as shown in Figure 2. When the train is moving, the coil inductance varies periodically because of the tooth-slot structure. Thus, the relative position can be obtained by detecting the changing coil inductance [8,9].

This paper studies the electromagnetic modeling of the sensor analytically, which lays an essential foundation for the sensor design work. Nowadays, with the development of PC technology, complex electromagnetic modeling problems can be realized through numerical methods precisely and conveniently, whereas, analytical methods are complicated, time consuming, and may even have no solution. Usually, in order to get a solution, model simplification is needed, which decreases the precision of the solution. However, analytical methods can get the expression of the field distribution function, represent less of a calculation load for computers, and are beneficial for the development of theories to some extent. They can be used to do some preliminary analysis and get a rough estimate for a problem to guide the principle design work. Numerical methods can be introduced later to guide the detailed design and parameter optimization.

Usually, it's difficult to solve 3-D electromagnetic problems with complex boundaries directly through separation of variables or mirror methods, *etc.* So, many researches are focused on 3-D field problems which can be converted to 2-D ones. Besides, second-order vector potential (SOVP) [10,11] can be introduced in to simplify vector field problems to scalar field ones. Reference [10] studies the electromagnetic field in an electrically conductive right-angled wedge due to an inductive excitation by a coil in air. The problem is simplified to a 2-D one. The magnetic induction intensity is expressed by a second-order vector potential (SOVP). The scalar components of the SOVP are expressed in terms of series expansions through Fourier transform. Reference [12] studies the 3-D Eddy-Current modeling problem with a spherical boundary in a spherical coordinate system. References [13–15] propose a semi-analytical method called Multipole Theory (MT). Its solution procedure and expressions are similar to those in reference [12], but this method is based on refined theoretical derivations, and can be applied to electromagnetic modeling problems with complicated boundaries by introducing New Equivalent Sources (NES).

The electromagnetic modeling of the relative position detection sensor is studied analytically in this paper. Firstly, the electromagnetic field in air due to an inductive excitation by a rectangular coil is calculated and analyzed. Based on this, a simplified model of the sensor is established. Then the magnetic induction density distribution of the simplified model is calculated semi-analytically according to the MT and NES methods, and a corresponding numerical solution is obtained through Finite Element Analysis (FEA). The comparison results show that the semi-analytical solution approximately agrees with the numerical solution. Finally, based on the modeling, a difference coil structure is designed to improve the sensitivity and precision of the sensor.

## 2. Sensor Model Simplification

### 2.1. Electromagnetic Field in Free Space Due to a Rectangular Coil

**Figure 3.**
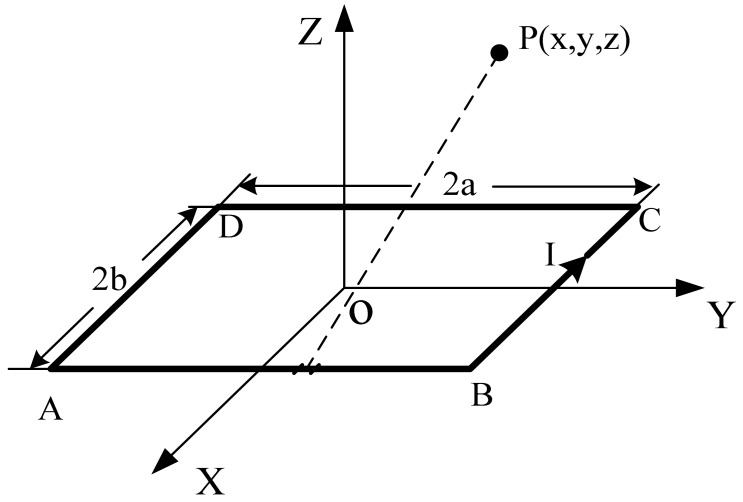
A rectangular coil in a Cartesian coordinate system.

The excitation frequency of the detecting coil is chosen to be about 2 MHz. The corresponding wavelength is *λ* = *v*/*f* = 150 m, which is much larger than the length of a tooth-slot period (86 mm). Thus, it can be considered a static magnetic field. Considering an ideal rectangular coil with current *I* and turn number *N* shown in Figure 3, according to Biot-Savart Law, the magnetic induction intensity at an arbitrary position *P*(*x,y,z*) due to each side of the coil is calculated respectively as follows [16–18]:

(1) $${\vec{B}}_{\text{AB}}=\frac{\mu_{0}\text{NI}\left[z\vec{j}-\left(y+b\right)\vec{k}\right]}{4\pi \left[{\left(y+b\right)}^{2}+z^{2}\right]}\left(\sin\beta_{1}-\sin\alpha_{1}\right)$$

where: $\alpha_{1}=tg^{-1}\frac{-\left(a+x\right)}{\sqrt{{\left(y+b\right)}^{2}+z^{2}}}$, $\beta_{1}=tg^{-1}\frac{a-x}{\sqrt{{\left(y+b\right)}^{2}+z^{2}}}$;

(2) $${\vec{B}}_{\text{BC}}=\frac{\mu_{0}\text{NI}\left[\left(x-a\right)\vec{k}-z\vec{i}\right]}{4\pi \left[{\left(x-a\right)}^{2}+z^{2}\right]}\left(\sin\beta_{2}-\sin\alpha_{2}\right)$$

where: $\alpha_{2}=tg^{-1}\frac{-\left(b+y\right)}{\sqrt{{\left(x-a\right)}^{2}+z^{2}}}$, $\beta_{2}=tg^{-1}\frac{b-y}{\sqrt{{\left(x-a\right)}^{2}+z^{2}}}$;

(3) $${\vec{B}}_{DC}=\frac{\mu_{0}\text{NI}\left[z\vec{j}-\left(y-b\right)\vec{k}\right]}{4\pi \left[{\left(y-b\right)}^{2}+z^{2}\right]}\left(\sin\beta_{3}-\sin\alpha_{3}\right)$$

where: $\alpha_{3}=tg^{-1}\frac{a-x}{\sqrt{{\left(y-b\right)}^{2}+z^{2}}}$, $\beta_{3}=tg^{-1}\frac{-\left(a+x\right)}{\sqrt{{\left(y-b\right)}^{2}+z^{2}}}$;

(4) $${\vec{B}}_{AD}=\frac{\mu_{0}\text{NI}\left[\left(x+a\right)\vec{k}-z\vec{i}\right]}{4\pi \left[{\left(x+a\right)}^{2}+z^{2}\right]}\left(\sin\beta_{4}-\sin\alpha_{4}\right)$$

where: $\alpha_{4}=tg^{-1}\frac{b-y}{\sqrt{{\left(x+a\right)}^{2}+z^{2}}}$, $\beta_{4}=tg^{-1}\frac{-\left(b+y\right)}{\sqrt{{\left(x+a\right)}^{2}+z^{2}}}$, and *i⃑, j⃑* and *k⃑* denote the unit vectors of coordinate axes X, Y and Z respectively.

According to the electromagnetic field superposition principle, the magnetic induction intensity in free space due to the rectangular coil is obtained as follows:

(5) $$\vec{B_{c}}=\vec{B_{\text{AB}}}+\vec{B_{\text{BC}}}+\vec{B_{\text{CD}}}+\vec{B_{\text{DA}}}$$

### 2.2. Sensor Model Simplification

**Figure 4.**
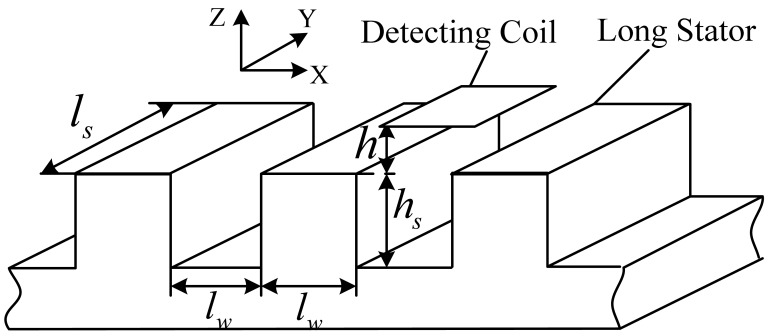
The coil-stator system.

The coil-stator system model is shown in Figure 4.

The length and width of the coil and the width of a slot or a tooth denoted by *l_w_* are all 43 mm, the depth of a slot is about *h_s_* = 50 mm, and the width of the long stator is about *l_s_* = 100 mm. Under normal working conditions, the distance between the coil plane and tooth plane is *h* = 8 mm.

**Figure 5.**
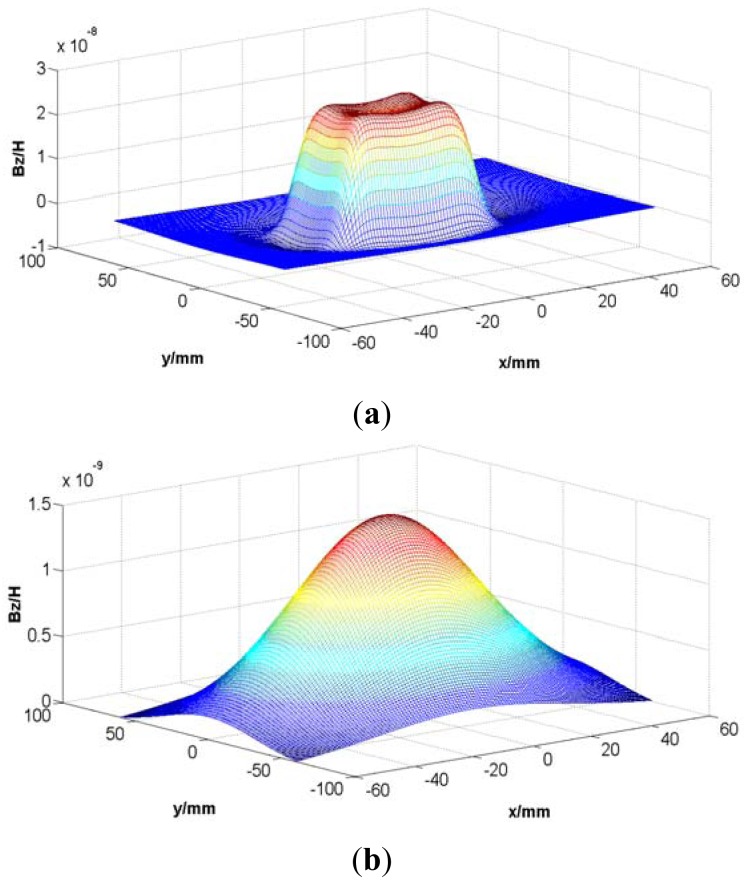
The Z-direction component of the magnetic induction intensity due to the rectangular coil excitation at (**a**) the tooth plane and (**b**) the slot plane.

The model is too complex for an analytical solution of the field, so some reasonable simplification is needed. Supposing *I* = 1 A and *N* = 1, according to Equations (1) to (5), the Z-direction component of the magnetic induction intensity at the tooth plane and the slot plane due to the rectangular coil excitation are simulated as shown in Figure 5.

**Figure 6.**
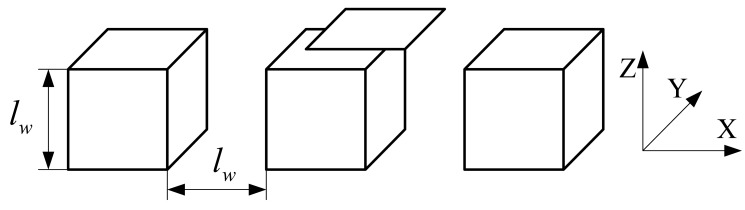
Simplified model of the coil-stator system.

According to Figure 5, the magnetic field is mainly distributed near the area facing the coil, and the magnetic induction intensity decreases rapidly with the increasing distance between the field plane and the coil plane, so the long stator shown in Figure 4 can be simplified to a series of cubes with an edge length *l_w_* = 43 mm shown in Figure 6.

**Figure 7.**
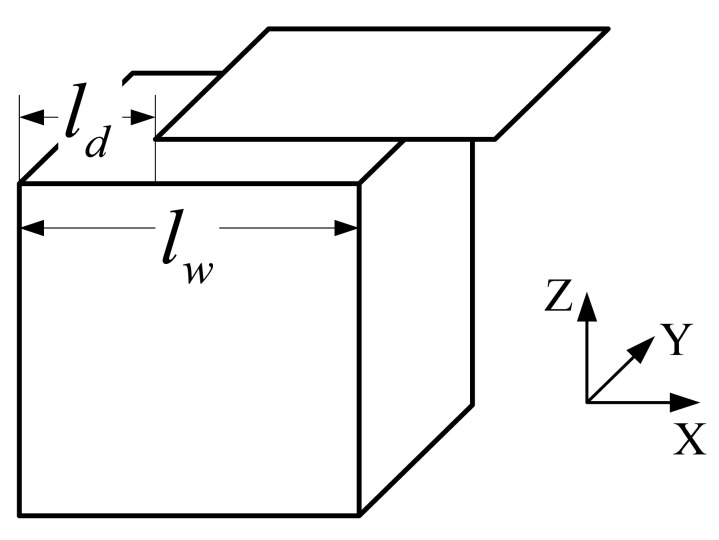
The final simplified model of the sensor.

Furthermore, considering the periodicity, the coil inductance is figured out only when 0< *l_d_* < *l_w_* shown in Figure 7. That's to say, the simplified model only consists of a cube and a square coil. In addition, the magnetic field due to the excitation coils is relatively weak, so the cube made of laminated silicon steel is regarded to be working in the linear region.

## 3. Electromagnetic Modeling of the Sensor

### 3.1. Multipole Theory for 3-D Static Magnetic Field

In a 3-D static field comprising several domains separated by medium boundaries, there exists a certain scalar *W*, such as electric potential or scalar magnetic potential, satisfying the Laplace equation:

(6) $$\nabla^{2}W=2$$

The domain in which *W* is to be solved is called the valid domain. In order to get the solution of *W* in this domain, auxiliary source points should be introduced into the field according to MT. An auxiliary source point set in the valid domain is called an inside pole. Otherwise, the pole is called an outside pole. For an open valid domain, there is no inside pole. For a finite valid domain, at most one inside pole can be set in it, and the pole had better be set at the geometrical center of the domain to make its corresponding series expansion converge fast [10].

The outside poles are set according to the boundaries. If all the inner angles between adjacent boundaries of the valid domain are less than 180°, each boundary should be assigned to an outside pole. For a spherical boundary surface, the pole should be set at the centre of the spherical surface to make the series expansion converge fast. For a plane boundary, it can be treated as a part of a spherical surface with an infinite radius. In this case, the series expansion corresponding to the plane boundary is reduced to a constant. Furthermore, if there exist inner angles greater than 180°, *W* can't be solved based on MT. In this case, the NES method [10] is introduced to solve the field approximately. The NES method will be discussed in the following section. The requirement of the inner angles of boundaries is called the expansion condition in MT.

**Figure 8.**
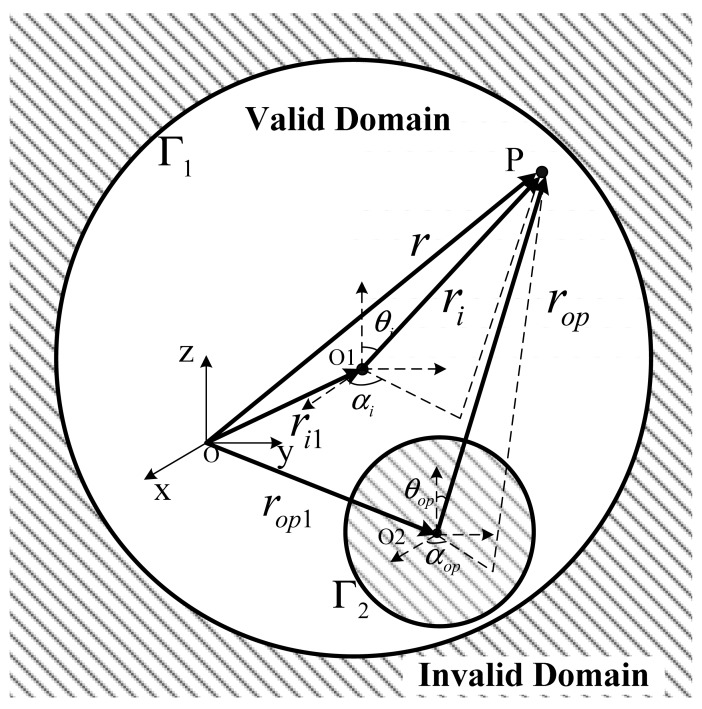
A field with two spherical boundaries.

Consider the situation shown in Figure 8. The inside pole is set at the centre of the valid domain denoted by *O*_1_. The outside pole is set at *O*_2_, the centre of spherical surface Γ_2_. The solution to the Laplace Equation (6) in the valid domain is given as follows [4,7,8]:

(7) $$\begin{array}{ll} W & =A+\sum_{l=1}^{N_{l}}r_{i}^{l}(A_{i}^{l}P_{l}(\cos\theta_{i})+\sum_{m=1}^{l}P_{l}^{m}(\cos\theta_{i})(A_{i}^{\text{lm}}\cos m\alpha_{i}+B_{i}^{\text{lm}}\sin m\alpha_{i}))+\sum_{p=1}^{N_{p}}[\frac{A_{\text{op}}}{r_{\text{op}}}+\sum_{l=1}^{N_{\text{op}}}r_{\text{op}}^{-(l+1)}(A_{\text{op}}^{l}P_{l}(\cos\theta_{\text{op}})+\sum_{m=1}^{l}P_{l}^{m}(\cos\theta_{i})(A_{\text{op}}^{\text{lm}}\cos m\alpha_{\text{op}}+B_{\text{op}}^{\text{lm}}\sin m\alpha_{\text{op}}))\\ & =\sum_{k=1}^{N}\alpha_{k}U_{k} \end{array}$$

where $r_{i}=\left|\vec{r}-\vec{r_{i1}}\right|$, $\vec{r_{i1}}$ is the position vector of the inside pole; $r_{\text{op}}=\left|\vec{r}-\vec{r_{\text{op}n}}\right|$, $\vec{r_{\text{op}n}}$ is the position vector of the *P* th outside pole; *N_i_* is the order of the inside pole ; *N_p_* is the number of the outside poles (in Figure 8, *N_p_* = 1); *N_op_* is the order of the *P* th outside pole; *N* is the total number of the terms of the series expression; *A*, $A_{i}^{l}$, $A_{i}^{\text{lm}}$, $B_{i}^{\text{lm}}$, $A_{\text{op}}$, $A_{\text{op}}^{l}$, $A_{\text{op}}^{\text{lm}}$ are coefficients determined by boundary conditions; *P_l_*(cos*θ_i_*) is Legendre polynomial and *P_l_^m^*(cos*θ_i_*) is associated Legendre function. *a_i_* and *θ_i_* are coordinates of field point *P* in spherical coordinate system *O*_1_. *a_op_* and *θ_op_* are coordinates of field point *P* in spherical coordinate system of the *p*th outside pole (in Figure 8, it is *O*_2_).

Experientially, to get a precise enough solution, the order of a pole corresponding to a spherical surface boundary can be set to 6, and the order of an inside pole corresponding to a cubic domain can be set to 10.

### 3.2. New Equivalent Source Method

#### 3.2.1. Spherical Equivalent Source Method

**Figure 9.**
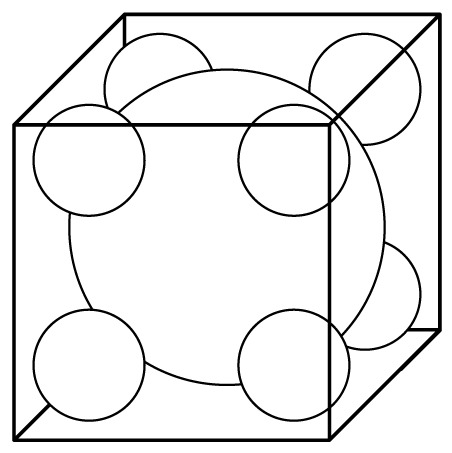
Spherical equivalent source.

Suppose the domain to be solved is the space outside the cube shown in Figure 9. The inner angle between adjacent boundaries is 270° dissatisfying the expansion conditions. In order to apply the MT method, we fill the cube with auxiliary spheres and treat these spherical surfaces as the boundaries instead of the surface of the cube. After this approximate substitution, MT can be applied to the problem. The substituted boundaries could be more approximate to the cube, if the cube is filled with more spheres. But increasing the number of the spheres will complicate the solution procedure.

#### 3.2.2. Circular Equivalent Source Method

**Figure 10.**
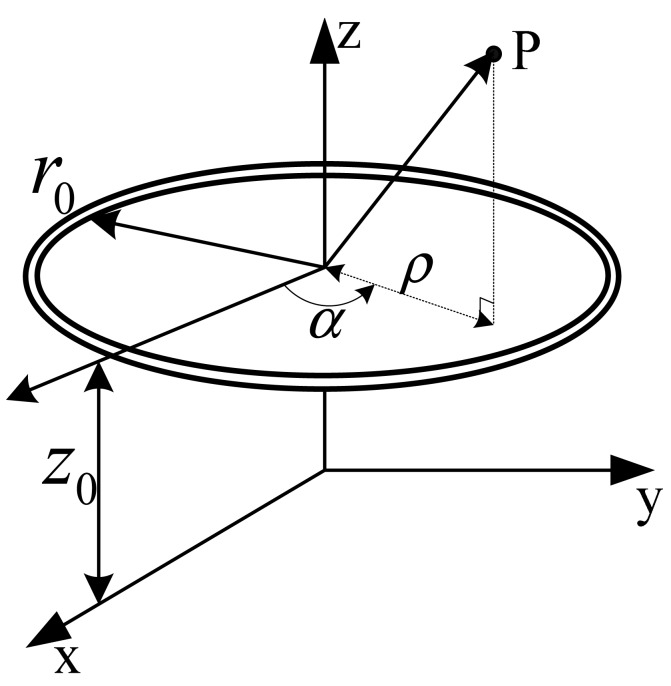
Circular equivalent source.

A circular equivalent source is shown in Figure 10. The scalar magnetic potential Ù at an arbitrary position *P*(*x,y,z*) due to the source satisfies Laplace equation, and has a solution given as follows [10]:

(8) $$\Omega =\frac{1}{2\pi \mu_{0}}\sqrt{\frac{r_{0}}{\rho }}\sum_{k=0}^{m_{c}}Q_{k-1/2}(Y)(c_{k}\cos k\alpha +d_{k}\sin k\alpha )$$

where *c_k_* and *d_k_* are undetermined coefficients; *Y*=((*z-z*_0_)^2^+*r*_0_^2^+*ρ*^2^)/2*r*_0_*ρ*; and *Q_k_*_-1/2_ is half-integer degree Legendre function [19], which can be expressed by the hypergeometric function *F*(*a,b,c,z*) as *Q_l_*(*t*)= *F*(*-l,l*+*1*,1,(1-*t*)/2) [14].

Thus, similar to the spherical equivalent sources, the invalid domain can be filled with auxiliary circular sources to replace the actual boundaries.

### 3.3. Semi-Analytical Solution of the Electromagnetic Field of the Simplified Sensor Model

Usually, it is hard to get the solution of a 3-D electromagnetic field problem by solving the Maxwell's equations directly, so Smythe [20] proposed a second-order vector potential (SOVP) *W⃗*, which can be expressed as the sum of two components normal to each other:

(9) $$\vec{W}=\vec{e}W_{1}+\vec{e}\times \nabla W_{2}$$

where e⃗ denotes i⃗, j⃗, k⃗ or $\vec{r}=r\vec{e_{r}}$; *W*_1_ and *W*_2_ are scalar components. The vector potential *A⃗* can be expressed as the curl of *W⃗*:

(10) $$\vec{A}=\nabla \times \vec{W}$$

In 3-D passive static electromagnetic field, the vector potential *W⃗* satisfies the Helmholtz equation:

(11) $$\nabla^{2}\vec{A}+k^{2}\vec{A}=0$$

where *k*^2^ = −j*ωμσ, ω* denotes the angular frequency of the field, *σ* denotes the electric conductivity and *μ* denotes the magnetic permeability. Considering the long stator of the high speed maglev train is made of laminated silicon steel, its eddy current is weak enough to be ignored, so *σ* can be regarded to be 0, and Equation (11) is reduced to a Laplace equation.

According to Equations (9–11), it can be concluded that *W*_1_ and *W*_2_ satisfy the Laplace equations:

(12) $$\nabla^{2}W_{1}=0$$

(13) $$\nabla^{2}W_{2}=0$$

The magnetic induction intensity *B⃗* satisfies:

(14) $$\vec{B}=\nabla \times \vec{A}=\nabla \times \nabla \times \vec{W}$$

Formula (14) results in the following expressions in spherical coordinate system:

(15) $$\left\{ \begin{array}{l} B_{r}=r\frac{\partial^{2}W_{1}}{\partial r^{2}}+2\frac{\partial W_{1}}{\partial r}\\ B_{\theta }=\frac{\partial^{2}W_{1}}{\partial r\partial \theta }+\frac{1}{r}\frac{\partial W_{1}}{\partial \theta }\\ B_{\varphi }=\frac{1}{\sin\theta }\frac{\partial^{2}W_{1}}{\partial r\partial \varphi }+\frac{1}{r\sin\theta }\frac{\partial W_{1}}{\partial \varphi } \end{array}\right.$$

According to Equation (15), *B⃗* is only a function of *W*_1_ which satisfies the Laplace Equation (12) and can be solved through MT and the NES method discussed above.

**Figure 11.**
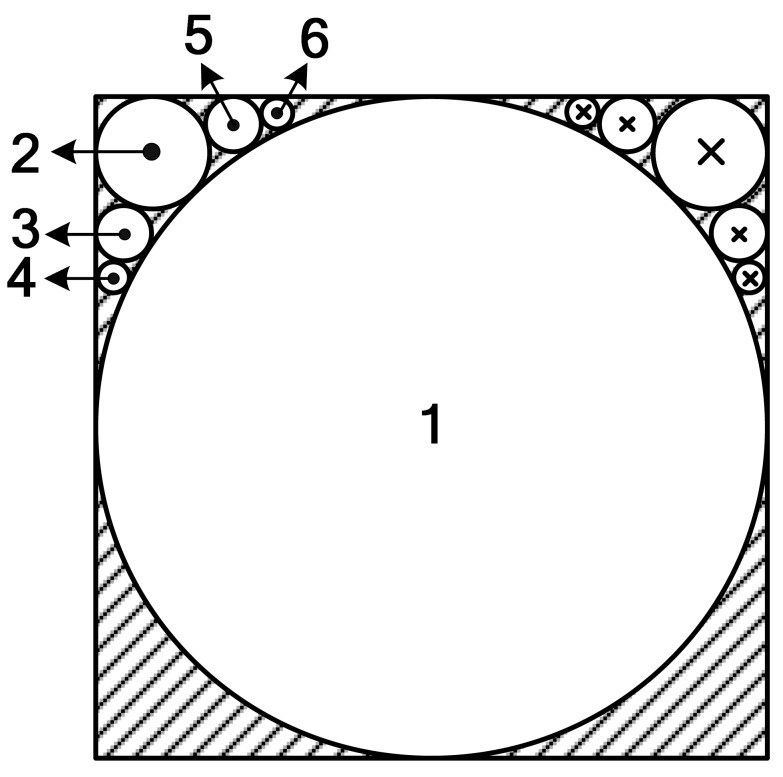
Configuration of the equivalent sources.

For the simplified problem model shown in Figure 7, there are two domains: the air domain (denoted by (I) and the silicon steel domain (denoted by II). The scalar components of SOVP in domain I and II denoted by *W*_I1_ and *W*_II1_ are calculated as follows, respectively:

1. Domain I
  In domain I, there exists an excitation coil, so the vector potential $\vec{A_{\text{I}}}$ satisfies the Possion equation:
  (16) $$\nabla^{2}\vec{A_{\text{I}}}=-\mu_{0}\vec{J}$$
  where *J⃗* is the current density of the coil. $\vec{A_{\text{I}}}$ is the sum of a particular solution $\vec{A_{\text{I}c}}$ of Equation (16) and the solution of the Laplace equation about $\vec{A_{\text{I}0}}$, so the magnetic induction intensity *B⃗_I_* can be expressed as:
  (17) $$\vec{B_{\text{I}}}=\vec{B_{\text{I}0}}+\vec{B_{\text{I}c}}$$
  The induction intensity due to the excitation coil in the free space can serve as a particular solution as $\vec{B_{\text{Ic}}}$, which is achieved in Equation (5).
  Considering domain I as the valid domain and according to the analysis above, it does not satisfy the expansion condition, so equivalent sources are introduced to replace the cubic boundary. The sectional view of the arrangement of equivalent sources in the cube is shown in Figure 11.
  The cube is filled with six equivalent sources. Source no. 1 is a spherical equivalent source and sources no. 2, 3, 4, 5 and 6 are circular equivalent sources. To calculate the inductance of the coil above the cube, the magnetic induction intensity near the upper surface of the cube should be precise enough, so equivalent sources are mainly set at the upper part of the cube to make the upper surface of equivalent sources more approximate to the upper plane of the cube. Because domain I is an open domain, there is no inside pole.
  Let *W*_I1_*_i_* denote the SOVP scalar component due to the *i* th source. According to the discussion above, we have:
  (18) $$W_{\text{I}11}=\frac{A_{\text{op}}}{r_{\text{op}}}+\sum_{l=1}^{N_{\text{op}}}r_{\text{op}}^{-(l+1)}(A_{\text{op}}^{l}P_{l}(\cos\theta_{\text{op}})+\sum_{m=1}^{l}P_{l}^{m}(\cos\theta_{i})(A_{\text{op}}^{\text{lm}}\cos m\alpha_{\text{op}}+B_{\text{op}}^{\text{lm}}\sin m\alpha_{\text{op}}))$$
  (19) $$W_{\text{I}12}+W_{\text{I}13}+W_{\text{I}14}+W_{\text{I}15}+W_{\text{I}16}=\sum_{i=1}^{5}\frac{1}{2\pi \mu_{0}}\sqrt{\frac{r_{0i}}{\rho }}\sum_{k=0}^{m_{c}}Q_{k-1/2}(Y_{i})(c_{k}\cos k\alpha_{i}+d_{k}\sin k\alpha_{i})$$
  As shown in Figure 7, the coil is moving along the X-axis, and the model is symmetrical referring to the X-O-Z plane, so the undetermined coefficients of the sine terms about *α* in Equations (18) and (19) are 0. Thus, *W*_I1_ can be expressed as follows:
  (20) $$W_{\text{I}1}=W_{\text{I}11}+W_{\text{I}12}+W_{\text{I}13}+W_{\text{I}14}+W_{\text{I}15}+W_{\text{I}16}=\frac{A_{\text{op}}}{r_{\text{op}}}+\sum_{l=1}^{N_{\text{op}}}r_{\text{op}}^{-(l+1)}(A_{\text{op}}^{l}P_{l}(\cos\theta_{\text{op}})+\sum_{m=1}^{l}P_{l}^{m}(\cos\theta_{i})A_{\text{op}}^{\text{lm}}\cos m\alpha_{\text{op}})+\sum_{i=1}^{5}\frac{1}{2\pi \mu_{0}}\sqrt{\frac{r_{0i}}{\rho }}\sum_{k=0}^{m_{c}}Q_{k-1/2}(Y_{i})c_{k}\cos k\alpha_{i}=\sum_{i=1}^{N_{1}}a_{i}U_{1i}$$
  where *a_i_* (*i* = 1…*N*_1_) are undetermined coefficients, and *N*_1_ is the term number. The meanings of other symbols are the same as in Equations (7) and (8). By substituting Equation (20) into Equation (15), the series expansion of $\vec{B_{\text{I}0}}$ can be obtained.
2. Domain II
  There is no excitation source in domain II, so the vector potential $\vec{A_{\text{II}}}$ satisfies the Laplace equation. The inner angles of the boundaries of domain II are 90° satisfying the expansion condition, and according to Section 3.1, the series expansions of the plane boundaries are reduced to constants, so there is only a series expansion of the inside pole set at the centre of the cube in this paper. Thus, *W*_II1_ can be expressed according to Equation (7) as follows:
  (21) $$W_{\text{II}1}=A+\sum_{l=1}^{N_{l}}r_{i}^{l}[A_{i}^{l}P_{l}(\cos\theta_{i})+\sum_{m=1}^{l}P_{l}^{m}(\cos\theta_{i})A_{i}^{\text{lm}}\cos m\alpha_{i}]=\sum_{i=1}^{N_{2}}b_{i}U_{2i}$$
  Where *b_i_* (*i* = 1…*N*_2_) are undetermined coefficients, *N*_2_ is the term number. By substituting Equation (21) into Equation (15), the series expression of $\vec{B_{\text{II}}}$ can be obtained.
3. Coefficients Determination
  The undetermined coefficients of Equations (20) and (21) can be determined by the Least Square (LS) method based on the boundary conditions. The boundary conditions between the air domain and the silicon steel domain are given as follows:
  (22) $$\left\{ \begin{array}{l} B_{\text{I}n}=B_{\text{II}n}\\ B_{\text{I}t1}/\mu_{0}=B_{\text{II}t1}/\mu \\ B_{\text{I}t2}/\mu_{0}=B_{\text{II}t2}/\mu \end{array}\right.$$
  where *μ* (about 5,600 *μ*_0_) denotes the permeability of silicon steel, *B*_I_*_n_, B*_II_*_n_* and *B*_I_*_t_*_1_, *B*_II_*_t_*_1_, *B*_I_*_t_*_2_, *B*_II_*_t_*_2_ denote the normal components and tangential components of the magnetic induction intensity, respectively. The LS method is adopted to minimize the error between the solutions of the two domains on the boundary. The target function is given as follows:
  (23) $$I_{LS}=\int_{\Gamma }[{\left(B_{\text{I}n}-B_{\text{II}n}\right)}^{2}+{\left(B_{\text{I}t1}-\frac{\mu_{0}}{\mu }B_{\text{II}t1}\right)}^{2}+{\left(B_{\text{I}t2}-\frac{\mu_{0}}{\mu }B_{\text{II}t2}\right)}^{2}]ds$$
  Where *I_LS_* is the function of the undetermined coefficients *a_i_* (*i* = 1…*N*_1_) and *b_i_* (*i* = 1…*N*_2_). In order to minimize *I_LS_*, let the partial derivatives of *I_LS_* about the undetermined coefficients equal to 0:
  (24) $$\{\begin{array}{cc} \frac{\partial I_{LS}}{\partial a_{i}}=0 & i=1,2\ldots N_{1}\\ \frac{\partial I_{LS}}{\partial b_{j}}=0 & j=1,2\ldots N_{2} \end{array}$$
  There are (*N*_1_ + *N*_2_) equations in Equation (24), so the (*N*_1_ + *N*_2_) undetermined coefficients can be determined. After substituting the coefficients *a_i_* (*i* = 1…*N*_1_) in the expression of $\vec{B_{\text{I}}}$, the coil inductance *L* can be obtained as:
  (25) $$L=\frac{\Phi }{I}=\frac{1}{I}\int_{S}\vec{B_{\text{I}}}\cdot d\vec{S}$$
  where *S* denotes the area of the coil.

## 4. Comparison between the Solutions of Semi-Analytical Method and Numerical Method

### 4.1. Simulation Model of the Numerical Method

**Figure 12.**
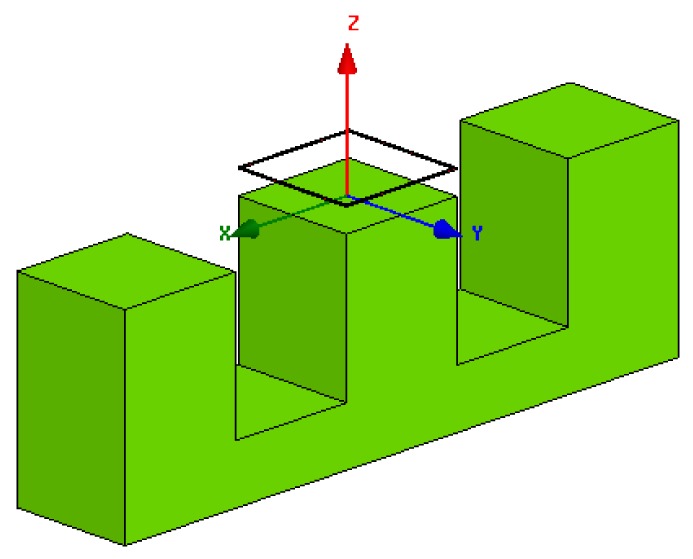
Simulation model of the numerical method.

The Finite Element Analysis (FEA) software Ansoft Maxwell is used to simulate the electromagnetic field distribution of the relative position detection sensor. The simulation model is shown in Figure 12.

### 4.2. Results of the Semi-Analytical Method and the Numerical Method

**Figure 13.**
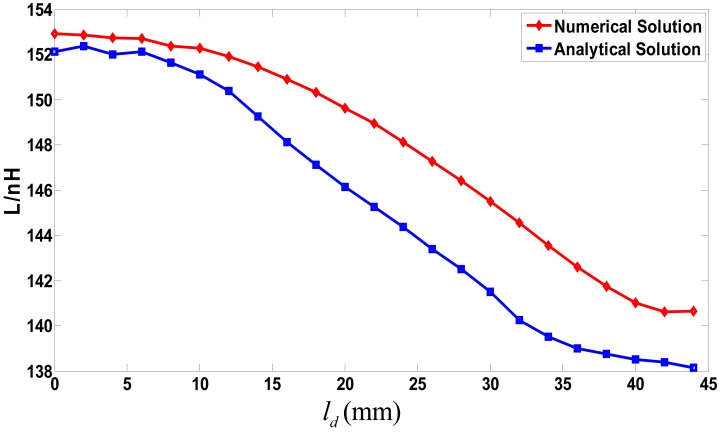
Relationship between coil inductance and relative position.

The calculation results of the coil inductance at different positions along the x-axis are shown in Figure 13, where the analytical modeling algorithm is implemented through MATLAB. Some special functions such as the Legendre polynomial and the hypergeometric function can be called straightforwardly from the MATLAB function library. According to Figure 13, there are errors between the numerical solution and the semi-analytical one. The maximal error is about 4.314 nH, which is much less than the coil inductance. The errors are mainly due to the model simplification and the discretization to get the undetermined coefficients by the LS method. In general, the coil inductance calculated through the semi-analytical method tends to approximately agree with that obtained by the numerical method, and the errors are within a tolerable limit.

## 5. Coil Design and Improvement

**Figure 14.**
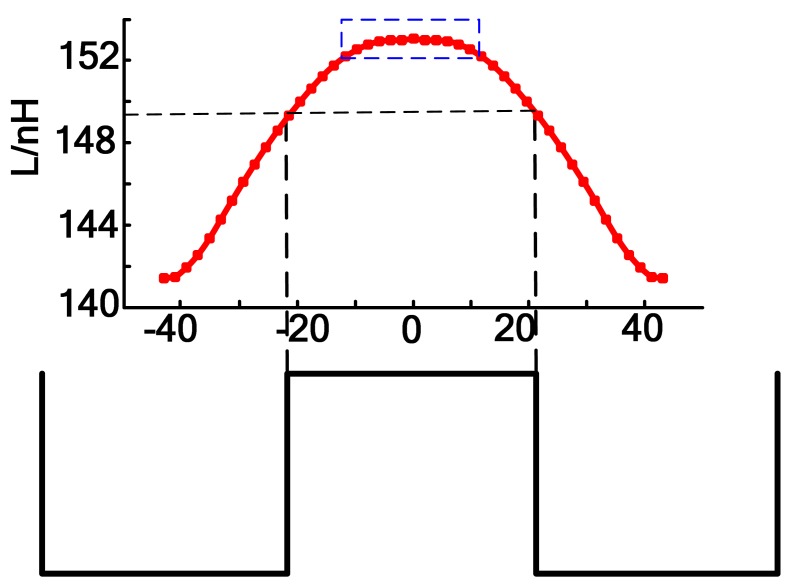
The relationship between coil inductance and the tooth-slot structure.

According the electromagnetic modeling results, the relationship between the coil inductance and the tooth-slot structure is shown in Figure 14. The relative position information can be gotten through table-lookup method according to this relationship.

**Figure 15.**
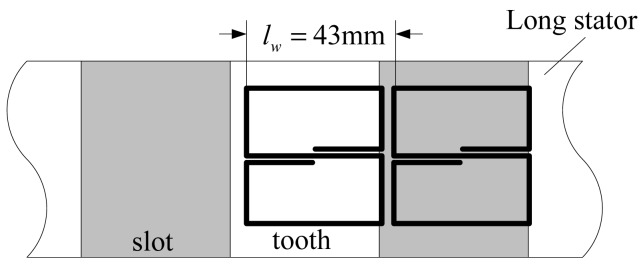
Difference coil structure.

**Figure 16.**
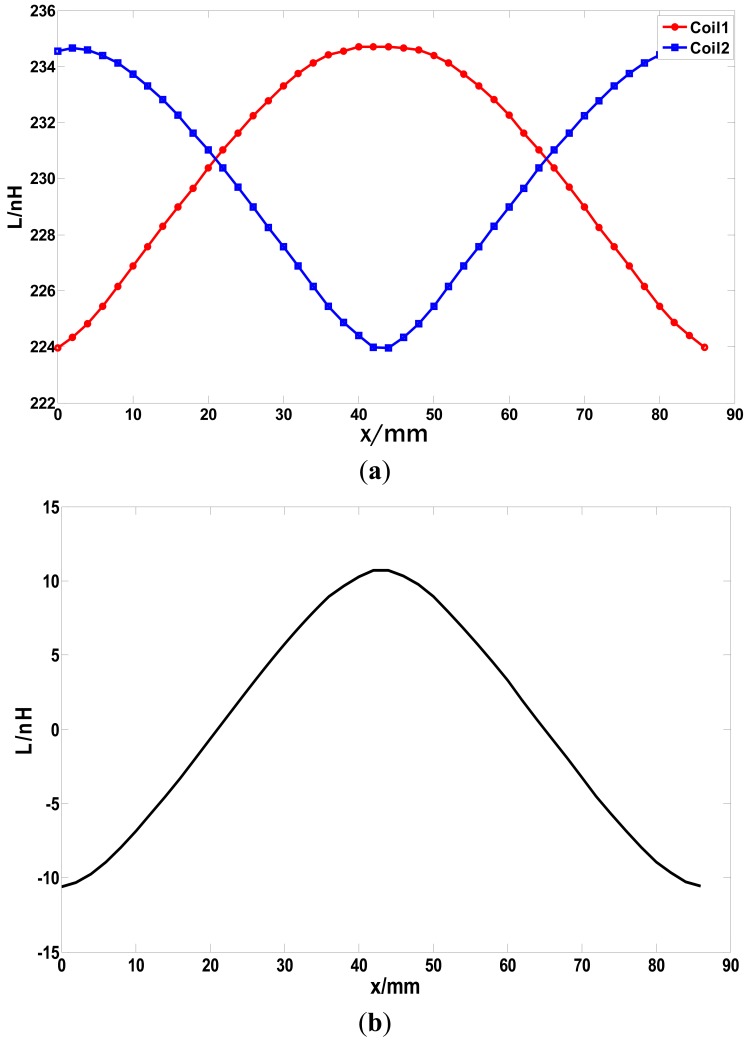
(**a**) Relative position and Inductance curve of coil 1 and 2; and (**b**) Inductance difference and relative position curve.

The curve shown in Figure 14 is similar to a sine wave but contains a large amount of harmonic components. When the coil is above a tooth, the slope ratio of the cure is small, which will reduce the sensitivity and accuracy of the table-lookup method. To improve this situation, a difference coil structure is proposed as shown in Figure 15. “8”-shaped Coils can counteract exterior electromagnetic disturbance. When the sensor is moving along the long stator at a certain constant speed, the relationship between the inductance of the two coils and the relevant displacement is shown in Figure 16(a). The phase difference of the two inductance curve is 180° because of the arrangement and dimension of the coils shown Figure 15. The difference inductance of the two coils has a better waveform as shown in Figure 16(b).

Thus, the precision of the sensor can be improved through the table-lookup method based on the relationship between the inductance difference and the relevant displacement. In addition, the difference operation can also counteract common mode disturbances such as temperature drift and enhance the performance of the sensor.

## 6. Conclusions

The relative position detection sensor obtains the relative displacement information between the train and the long stator by detecting the tooth-slot structure of the long stator based on nondestructive technology. This paper researches the electromagnetic field modeling of the sensor to guide the coil design and improvement. The electromagnetic field model of the coil-stator system is a complex three-dimensional (3-D) electromagnetic problem, so at first, the problem is simplified based on the analysis of the electromagnetic field distribution of a rectangular coil in free space. And then, MT and the NES method are adopted to get the series expansion of the magnetic induction intensity of the simplified model. The relationship between the coil inductance and the relative position is figured out afterwards. The inductance is also simulated through FEA. The comparison results show that the semi-analytical solution approximately agrees with the numerical solution. Finally, based on the modeling work, a difference coil structure is designed to improve the sensitivity and precision of the relative position detection sensor.
